# Is Ectopic Cushing Syndrome Commonly Associated with Small Cell Lung Cancer (SCLC)? Critical Review of the Literature and ACTH Expression in Resected SCLC

**DOI:** 10.1007/s12022-025-09860-5

**Published:** 2025-05-02

**Authors:** Ayako Ura, Elisa Moser, Matthias Evert, Katja Evert, Bruno Märkl, Eva Sipos, Marcus Kremer, Hironobu Sasano, Yoshinori Okada, Katja Steiger, Carolin Mogler, Hans Hoffmann, Alexander von Werder, Daniel Kaemmerer, Silvia Uccella, Stefano La Rosa, Günter Klöppel, Atsuko Kasajima

**Affiliations:** 1 Department of Pathology, Technical University of Munich, TUM School of Medicine and Health, Munich, Germany; 2 Institute of Pathology, University of Regensburg, Regensburg, Germany; 3 Pathology, Faculty of Medicine, University Augsburg, Augsburg, Germany; 4 Institute of Pathology, Städtisches Klinikum München, Munich, Germany; 5 Department of Pathology, Tohoku University Graduate School of Medicine, Sendai, Japan; 6 Department of Thoracic Surgery, Tohoku University Graduate School of Medicine, Sendai, Japan; 7 Department of Thoracic Surgery, Technical University of Munich, TUM School of Medicine and Health, Munich, Germany; 8 Department of Internal Medicine II, Technical University of Munich, TUM School of Medicine and Health, Munich, Germany; 9 Department of General and Visceral Surgery, Zentralklinik Bad Berka, Bad Berka, Germany; 10 Department of Biomedical Sciences, Pathology Unit, Humanitas University, Milan, Italy; 11 Pathology Service, IRCCS Humanitas Research Hospital, Rozzano, Italy; 12 Department of Medicine and Technological Innovation, University of Insubria, Varese, Italy

**Keywords:** Ectopic Cushing syndrome, Lung, SCLC, ACTH

## Abstract

The literature emphasizes that pulmonary ectopic Cushing syndrome (ECS) is associated not only with neuroendocrine tumors (NETs), but also with small cell lung carcinomas (SCLCs). This statement is debatable, because extrapulmonary ECS is associated with NETs in the vast majority of cases and very rarely with neuroendocrine carcinomas (NECs). Therefore, we critically reviewed the literature on SCLCs associated with ECS (ECS-SCLC) and performed immunohistochemical analysis of ACTH expression in 155 resected SCLCs and 158 pulmonary NETs. The literature search revealed that 90% of the 205 ECS-SCLC patients identified between 1952 and 2023 had no or poor-quality histologic images, so the diagnosis of SCLC could not be confirmed. Review of the 20 reports (10%) with histologic images revealed that 18/20 (90%) had to be reclassified as "probable NET", of which 5/18 (28%) showed spindle cell morphology, while only 2 cases were qualified as "SCLC compatible" due to their pleomorphic cell features. Immunohistochemically, 5/155 (3%) resected SCLCs, all without ECS, showed weak single cell ACTH expression, whereas in the NET cohort, 61/158 (39%) tumors expressed ACTH, of which 4 (3%) were associated with ECS. Both observations, the literature review, which casts doubt on previously reported data regarding the frequency of SCLC in ECS, and the immunohistochemical study, suggest that there is limited evidence that SCLC is the cause of ECS.

**Supplementary Information:**

The online version contains supplementary material available at 10.1007/s12022-025-09860-5.

## Introduction

Cushing syndrome, clinically characterized by obesity, hypertension, and hypokalemia, dates back to a study by Harvey Cushing in 1932 in which he found an association between the syndrome and a pituitary tumor [1]. The same symptoms had been reported by Brown in 1928 in a patient with small cell lung cancer (SCLC), then also called oat cell carcinoma of the lung [2]. Because Brown’s patient had an enlarged pituitary gland in addition to the lung tumor, the relationship between the patient’s syndrome and oat cell carcinoma could not be clearly established [2]. In 1952, Thorne et al. reported two patients with identical symptoms and bronchial oat cell carcinoma without pituitary enlargement [3], considered the first patients with ectopic Cushing syndrome (ECS) associated with SCLC. Adrenocorticotropic hormone (ACTH) secretion in non-pituitary tumors was demonstrated by radioimmunoassay in 1968 [4] and by immunohistochemistry in tumor tissue in 1975 [5]. To date, more than 750 patients with ECS have been recognized and reported, the majority (71–88%) with pulmonary neuroendocrine neoplasms (NENs). Rarely, ECS (2–25%) may also be associated with extrapulmonary NETs such as pancreatic neuroendocrine tumors (NETs) [6], medullary thyroid carcinoma or pheochromocytoma [7–13]. Pulmonary NENs in ECS include both NETs and neuroendocrine carcinomas (NECs), with a highly variable incidence between studies ranging from 8 to 92% for both NETs and NECs, according to seven clinical studies in ECS patients summarized in Supplementary Table 1 [7, 8, 10–13]. Of the reported pulmonary NECs causing ECS, almost all were SCLC, whereas large cell neuroendocrine carcinoma (LCNEC) causing ECS appears to be very rare [12, 14] (Supplementary Table 1). The percentage of SCLCs with ECS ranges from 0.2 to 4.8% [15–17], with only a few studies reporting a frequency between 12 and 19% [18, 19]. Almost all of these studies were performed between 1970 and 1993 [16, 18, 19] and did not include morphological evaluation.

The observations that the frequency of SCLCs associated with ECS is highly variable, coupled with the rarity of NECs associated with extrapulmonary ECS (see Supplementary Table 1) [20], raise questions about the relationship between ECS and SCLC and its documentation in literature, and need to be discussed. Therefore, the aim of this study is to determine the strength of evidence for an association of SCLC with ECS through a systematic review of the published reports. Second, we searched for ACTH expression in resected SCLCs and pulmonary NETs to determine whether ACTH-positive cells are a component of SCLCs.

## Materials and Methods

### Systematic Literature Review

As a first step, we searched PubMed for previously reported SCLC with ectopic Cushing syndrome using the following keywords: 1) organ (e.g., bronchus, lung), 2) tumor type (e.g., SCLC, oat cell carcinoma), and 3) functional parameters (e.g., adrenocorticotropin, ACTH, Cushing syndrome), combined with "AND". The specific terms used as keywords are listed in Supplementary Table 2. Literature that was not available online was copied from the university library of the Technical University of Munich, Germany. The remaining publications, which were not linked to PubMed, were identified through other search programs (including Google Scholar, the university and publisher archives, Ichushi or J-stage). We obtained 3991 articles and extracted clinical and histopathological features (sex, age, tumor size, smoking history, tumor extension, and outcome) of patients. Review articles, basic research studies without clinical data, clinical trials, epidemiological studies, letters to the editor, studies on other tumor entities, and articles written in languages other than English, German, or Japanese were excluded.

Pathology data important for classification of the tumor entity such as Ki67 index, mitotic count, presence or absence of necrosis and immunohistochemical findings were extracted from the text and available histologic, cytologic and electron microscopic images documented regarding staining (H&E, immunohistochemistry), color (colored or black and white) and number of images.

### Review of Diagnosis Based on Images

The provided histopathological images were reviewed by three pathologists (AU, GK and AK), including two experts for neuroendocrine neoplasms, and re-classified into two categories: “SCLC compatible” and “probable NET”. Distinction of SCLC from NET was based on a four-criteria-scoring of NET characteristics including uniformly round nuclei, abundant cytoplasm, nested organoid pattern, and delicate vasculature between the nests [21–23]. Each feature was scored absent (score 0) or present (score 1). If two or more NET features were seen, a score of 2 to 4 was given and the tumor classified as "probable NET", while tumors grouped as "SCLC compatible" showed none or only one of the NET features (score of 0 or 1). Spindle cell morphology was separately documented. Histologic images completely out of focus or had prominent crush artefacts were not evaluated.

### Assembling of Lung Tumor Tissue and Patients´ Information

For the immunohistochemical study of ACTH expression in lung tumors, two large cohorts from Germany (*n* = 132) and Japan (*n* = 181) were examined, comprising 155 resected primary SCLCs and 158 bronchopulmonary NETs. Surgery was performed at 15 different institutions between 1996 and 2024 (see Supplementary Table 3 for details). The clinical data of the patients, including age, gender, smoking status, and metastatic status, were obtained from the medical records. The absence or presence of ECS was meticulously reviewed. The patients with SCLC were not preoperatively monitored for serum ACTH levels. Patients with pulmonary NETs were also not monitored for serum ACTH levels, except for the four patients with ECS. Follow-up data were available for 110 SCLC patients (71%) and 139 NET patients (88%). Progression-free survival (PFS) was defined as the time between initial surgery and relapse or disease-related death. Overall survival (OS) was defined as the time from initial surgery to disease-related or unrelated death. This study was approved by the local ethics committees of the Technical University of Munich, Germany (2022–396-S-DFG-SR) and Tohoku University, Sendai, Japan (2018–1–515).

### Histologic and Immunohistochemical Evaluation

Formalin-fixed paraffin-embedded (FFPE) tissue blocks from representative tumor areas were utilized for further examinations. The histologic diagnosis was reviewed by three pathologists (see above) according to the WHO classification [21, 24] using 2-μm-thick sections stained for H&E and immunolabelled with cytokeratin 18, chromogranin A, synaptophysin, CD56, TTF- 1, Ki67, p53, retinoblastoma 1 (Rb1), somatostatin receptor 2 (SSTR2) and ACTH. All staining procedures were conducted using a fully automated system (Benchmark XT; Ventana/Roche, Arizona, USA). Further details regarding the immunohistochemical staining are provided in Supplemental Table 4. ACTH was evaluated using a four-grade scoring system – score 0 (no positive cells), score 1 (single cell < 10%), score 2 (10–50%), and score 3 (> 50%). Score 0 was regarded as negative and score 1, 2, and 3 as positive.

### Statistical Analysis

JMP Pro version 17.0.0 software (SAS Institute, Inc., Cary, NC, USA) was used for all statistical analyses. Pearson’s chi-squared test or Fisher´s exact test was used to compare sample sizes between groups. The Wilcoxon test was used to compare continuous values or scores between multiple groups that were not normally distributed according to the Shapiro–Wilk test. The probability of differences in PFS and DSS was determined using the Kaplan–Meier method, with a log-rank test for significance. A *p*-value of < 0.05 was considered statistically significant.

## Results

### Data Extracted From Reported Cases

A total of 99 articles published between 1952 and 2023 were identified, including 205 SCLC patients with ECS. Articles were published either as case reports presenting individual patients (occasionally two or three) or as cohorts including between 4 and 14 SCLC patients. Articles published prior to 1990 and using old terminologies, such as "oat cell carcinoma" for SCLC, were also included. The clinical characteristics of the reported patients are summarized in Supplementary Table 5.

Information important for re-evaluation of the histological diagnosis, in particular mitotic count, Ki67 labelling index or extent of necrosis, was only provided in one patient for mitotic count and in three patients for Ki67 (Supplementary Table 5).

**Fig. 1:**
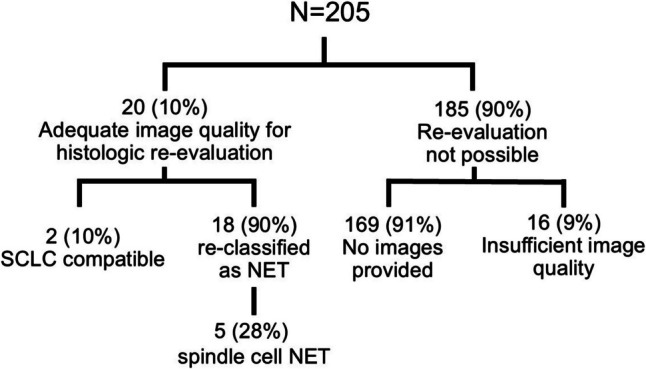
Availability of histologic images in 205 cases of reported small cell lung carcinoma (SCLC) associated with ectopic Cushing syndrome and results after re-evaluation of the histologic features in 20 cases

Only 20 of the 205 reported SCLC cases with ECS published between 1958 and 2021 included histologic and/or immunohistochemical images that could be used for reviewing the diagnosis. In further 16 case reports, the images were not sufficient to re-evaluate of the diagnosis, e.g., only one immunohistochemical image of ACTH and no H&E section (see Fig. 1). The majority of the reports (13/20) included one or two histologic images (see Table 1 for details). Color images were only provided in articles published after 2009 (Table 1).

**Table 1:** Number and type of provided histological images of reported small cell lung cancer cases associated with ectopic Cushing syndrome and results of re-evaluation of the diagnosis using a score for NET-characteristic features

ID	Year	PMID	Age sex	Number and type of images	Ki-67 LI (%)	NET characteristic features ^l^					Decision after re-evaluation
						Uniformly round or oval nuclei	Abundant cytoplasm	Nested organoid pattern	Delicate vasculature between the nests	Total score	
1	2021	33971870	64 M	5^a^ HE, IHC	80	Absent	Absent	Absent	Absent	0	SCLC compatible
2	2020	32492750	58 M	2^b^ HE, IHC	ND	Present	Present	-	-	2	probable NET
3	2019	31243230	72 M	3^c^ HE, IHC	ND	Present	Present	Present	Present	4	probable NET
4	2016	26875965	64 M	4^d^ HE, IHC	ND	Present	Present	Present	Present	4	probable NET
5	2014	24048720	59 M	2^e^ HE, IHC	ND	Present	-	Present	Present	3	probable NET spindle cell type
6	2010	21240327	60 F	4^f^ HE, Cytology	ND	Present	Present	Present	Present	4	probable NET
7	2010	ND	37 M	1 HE	ND	Present	Present	-	-	2	probable NET
8	2009	19829770	54 M	1 HE	ND	Absent	Absent	Absent	Present	1	SCLC compatible
9	2007	18018629	54 F	4^g^ HE, IHC	ND	Present	Present	Present	Present	4	probable NET
10	2007	17418447	61 F	2^h^ IHC	ND	Present	Absent	Present	Present	3	probable NET
11	1996	8622281	70 F	2^b^ HE, IHC	ND	Present	Present	Present	Present	4	probable NET spindle cell type
12	1993	8390589	62 M	1 HE^i^	ND	Present	Present	Present	Present	4	probable NET spindle cell type
13	1985	2417404	26 M	6^j^ HE, IHC, EM	ND	Present	Present	Present	Present	4	probable NET
14	1979	225397	57 F	2^k^ HE, EM	ND	Present	Present	Present	Present	4	probable NET spindle cell type
15	1977	ND	60 F	2^k^ HE, EM	ND	Present	Present	Present	Present	4	probable NET
16	1972	4342852	59 M	2 HE^i^	ND	Present	Present	Present	Present	4	probable NET
17	1961	ND	53 M	1 HE^i^	ND	Present	Present	Present	Present	4	probable NET
18	1960	14416539	41 M	2 HE^i^	ND	Present	Present	Present	Present	4	probable NET spindle cell type
19	1958	13600639	68 M	1 HE^i^	ND	Present	Present	Present	Present	4	probable NET
20	1958	13570643	22 M	1 HE^i^	ND	Present	Present	Present	Present	4	probable NET

*ACTH*, Adrenocorticotropic hormone; *HE*, Hematoxylin-Eosin staining; *BW*, black and white; *IHC*, Immunohistochemistry; *EM*, Electron microscopy; *CgA*, Chromogranin A; *TTF-1*, Thyroid transcription factor-1; *CRH*, Corticotropin releasing hormone; *NSE*, Neuron specific enolase; *ADH*, Antidiuretic hormone; *ND*, No data; *SCLC*, Small cell lung carcinoma; *NET*, Neuroendocrine tumor; *NEC*, Neuroendocrine carcinoma, a) 3 HE in color, Ki67, and ACTH, b) HE in color and ACTH, c) 2HE and ACTH, d) HE in color, ACTH, CRH, and γ3-MSH, e) HE in color and CD56, f) 2 HE in color and 2 cytology, g) 2 HE in BW, CgA, and TTF-1, h) ACTH and ADH, i) HE in BW, j) HE in BW, ACTH, salivary amylase, and 3 EM, k) HE in BW and EM, l) Absent findings were scored 0 and present findings were scored 1. The total score was the sum of the present findings

### Review of Histologic Images

Images of 20 SCLC cases were scored (see Table 1 for details). Of these, 18/20 tumors (90%) had a total score of 2 or higher and were therefore grouped as "probable NET"(ID2 - 7, 9–20). 5 of 20 (25%) tumors showed a spindle-cell histology and were classified as spindle cell NET (ID5, 11, 12, 14, 18) (Fig. 1). Mitoses could only be detected in 1 of the provided images (ID3), while no mitoses were seen in the other cases (ID2, 4, 6–9, 11–20). In two tumors, mitoses could not be recognized because of the low magnification (ID5, 10). None of the reports provided Ki67 index data. Information on the presence or absence of necrosis was not found, and necrosis was not detectable in any of the images.

The histologic images of two tumors (ID1 and ID8 in Table 1) revealed no NET-typical features and were classified as "SCLC compatible". The tumor ID1 showed a diffuse growth pattern of small cells with narrow cytoplasm accompanied by a large cell neuroendocrine carcinoma component with a high Ki67 index (80%). The histologic image of ID8 showed a cluster of pleomorphic tumor cells with narrow cytoplasm (Table 1, Fig. 1).

### Immunohistochemical Expression of ACTH and Clinicopathological Features in Resected SCLCs and NETs

Clinicopathological features of 155 SCLC and 158 NET patients are shown in supplementary Table 6.

**Fig. 2:**
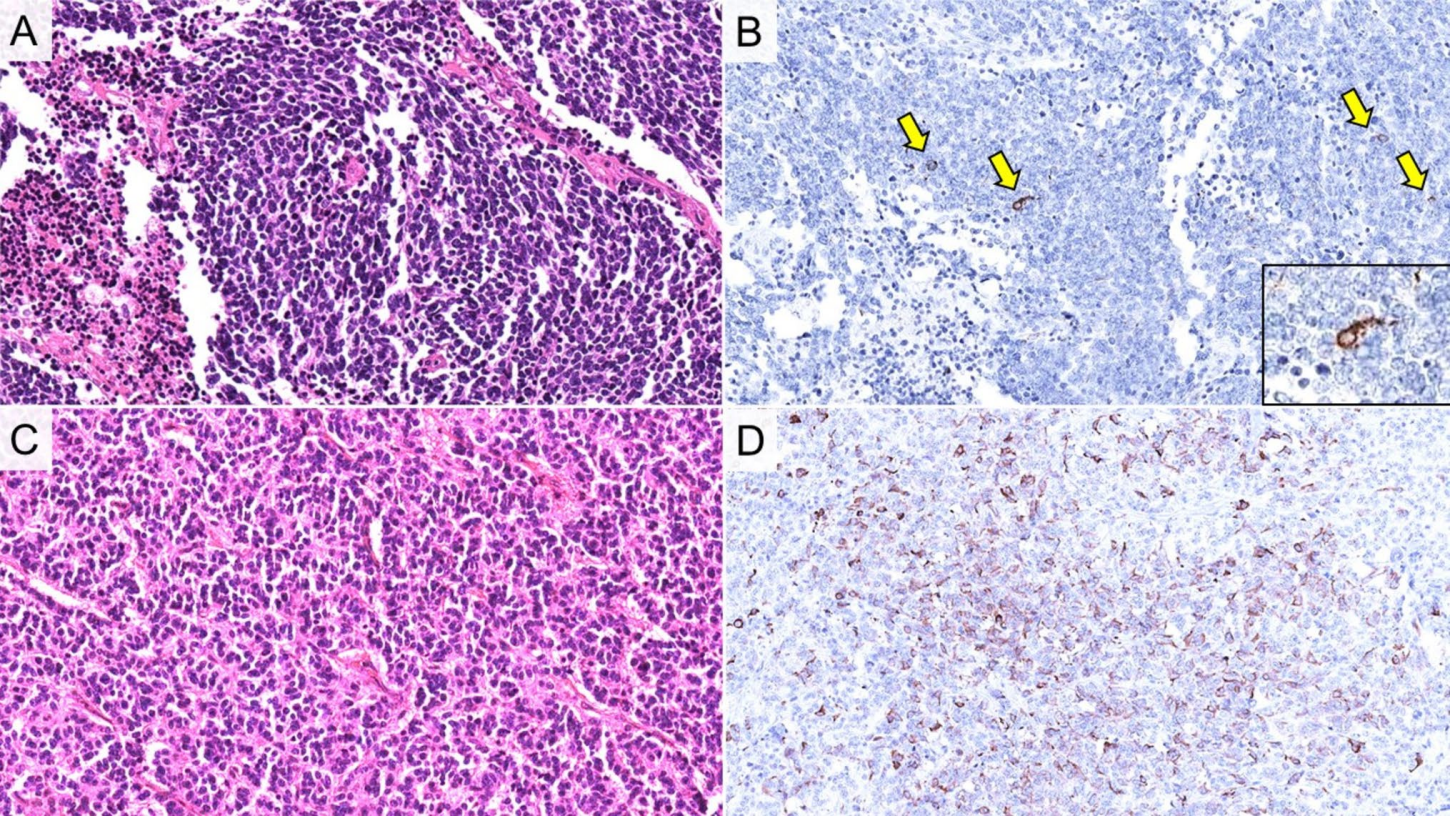
**A**) Histologic images and **B**) single cell ACTH expression in small cell lung carcinoma (SCLC). **C**) histologic image of pulmonary neuroendocrine tumor (NET)/carcinoid and **D**) diffuse ACTH positivity (score 3 +)

**Fig. 3:**
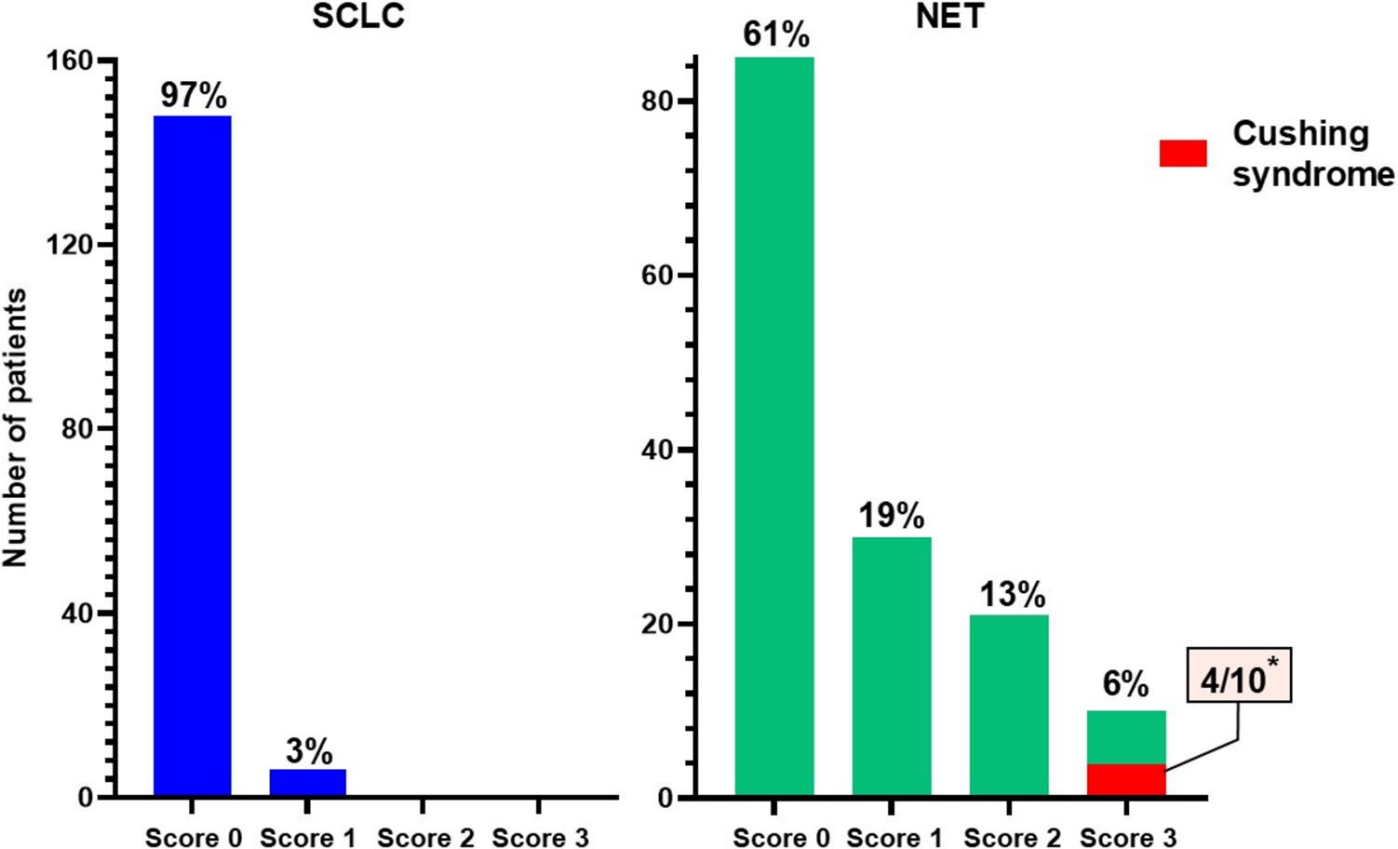
Immunohistochemical scores of ACTH expression in 155 resected small cell lung carcinomas (SCLCs, blue) and 158 resected pulmonary neuroendocrine tumors (NETs)/carcinoids (green). Patients with ectopic Cushing syndrome (ECS) was found in 4 out of 10 NET patients with score 3 + ACTH expression (asterisk)

Focal (< 5% of tumor cells) and weak single cell ACTH expression (score 1 +) was found in 5/155 resected SCLCs (3%, Fig. 2). In resected lung NETs, ACTH positivity was observed in 61/158 (39%) cases (19% score 1 +; 13% score 2 +; 6% score 3 +) (Fig. 2). Detailed review of medical records revealed that none of the SCLC patients had ECS, while ECS was found in 4/10 NET patients, all of whom had NETs with ACTH score 3 (supplementary Table 6, Fig. 3).

No statistical differences were observed between the clinicopathologic characteristics of ACTH-positive and negative SCLCs (Table 2). In particular, Ki67 status and p53 overexpression were not associated with ACTH expression. No statistical difference was observed in PFS or in OS between SCLC patients with and without ACTH expression (data not shown). ACTH expression in pulmonary NETs were seen in older patients (*p* = 0.03), more often in females (*p* < 0.0001), in never smokers (*p* = 0.02) than ACTH negative NETs and were seen more often in TCs (*p* = 0.02) and less frequently expressed SSTR2 (*p* < 0.0001) (Table 3). NET patients with ECS were younger than those without ECS (median age 63 vs. 73, *p* = 0.03) and were all females. No difference was found in smoking status, Ki67 index, p53, retinoblastoma, SSTR2 expression, or outcome (both PFS and OS) between NET patients with and without ECS (data not shown).

**Table 2:** Comparison of clinicopathological features between ACTH-negative and -positive resected small cell lung carcinomas

			ACTH expression		*p*-value
			Positive	Negative	
Total	N		5	150	
Age^a^		Median (range)	67 (44–78)	69 (43–86)	NS
Sex^a^	N (%)	M: F	3 (60): 2 (40)	117 (85): 21 (15)	NS
Smoking^b^	N (%)	Smoker	1 (100)	101 (97)	NS
		Never smoker	0	3 (3)	
Ki- 67 LI	%	Median (range)	47.5 (45–90)	63 (25–99)	NS
p53 status^c^	N (%)	Abnormal	5 (100)	79 (62)	NS
		Normal	0	49 (38)	
RB1 status^d^	N (%)	Abnormal	5 (100)	96 (88)	NS
		Normal	0	13 (12)	
SSTR2^e^	N (%)	Positive	3 (60)	42 (43)	NS
		Negative	2 (40)	56 (57)	
ACTH	N (%)	Score 0	0	150 (100)	-
		Score 1	5 (100)	0	
		Score 2	0	0	
		Score 3	0	0	
Cushing syndrome	N (%)	Absent	5 (100)	150 (100)	NS
		Present	0	0	

Abbreviations: *M* male, *F* female, *RB1* Retinoblastoma 1, *SSTR2* Somatostatin Receptor 2, *NS* not significant

Data missing in ^a^12 patients, ^b^50 patients, ^c^22 patients, ^d^41 patients, ^e^52 patients

**Table 3:** Comparison of clinicopathological features between ACTH-negative and -positive resected pulmonary neuroendocrine tumors

			ACTH expression		
			Positive	Negative	*p*-value
Total	N		61	97	
Age		Median (range)	70 (33–84)	64 (11–86)	0.03
Sex	N (%)	M: F	5 (8): 56 (92)	50 (52): 47 (48)	< 0.0001
Smoking^a^	N (%)	Smoker	6 (25)	26 (54)	0.02
		Never smoker	18 (75)	22 (46)	
Ki- 67 LI	%	Median (range)	2 (0.5–13)	2 (0.2–62)	NS
NET classification	N (%)	TC	53 (87)	71 (73)	0.04
		AC	8 (13)	26 (27)	
p53 status^b^	N (%)	Abnormal	0	0	-
		Normal	54 (100)	87 (100)	
RB1 status^c^	N (%)	Abnormal	1 (2)	6 (8)	NS
		Normal	50 (98)	74 (93)	
SSTR2^d^	N (%)	Positive	15 (29)	52 (68)	< 0.0001
		Negative	37 (71)	25 (32)	
ACTH	N (%)	Score 0	0	97 (100)	-
		Score 1	30 (49)	0	
		Score 2	21 (34)	0	
		Score 3	10 (16)	0	
Cushing syndrome	N (%)	Absent	57 (97)	97 (100)	0.01
		Present	4 (3)	0	

Abbreviations: *M* male, *F* female, *RB1* Retinoblastoma 1, *SSTR2* Somatostatin Receptor 2, *TC* typical carcinoid, *AC* atypical carcinoid, *NS* not significant

Data missing in ^a^86 patients, ^b^17 patients, ^c^27 patients, ^d^29 patients

## Discussion

Since its first description in 1952, ECS associated with SCLC has been reported in many studies. Consequently, SCLC is considered to be one of the most common neoplasms causing ECS. However, because of the rarity of extrapulmonary NECs with ECS [10, 20] and the highly variable number of SCLCs with ECS reported in different studies, we questioned the strong association between ECS and SCLC and performed a systematic literature review of all published cases found in PubMed and other search programs. We reviewed all reports of SCLC (including oat cell carcinoma) causing ECS, from the publication of the first case in 1952 [3] to 2023 [25]. In 205 patients from 99 publications, we found the description and diagnosis of an ECS that was attributed to an ACTH-secreting SCLC [26–28]. However, histological and immunohistological images of diagnostic quality to confirm this tumor diagnosis were found in only 20 publications. To further validate the histologic diagnosis of SCLC and especially to distinct SCLC from pulmonary NET (carcinoid), the main cause of ECS, we used a scoring system for the morphologic features of NET [23, 29]. We found that the histological images of 18 of the 20 reported cases (90%) did not show the typical features of SCLCs, i.e., sparse cytoplasm with poorly defined cell borders, small cell size (less than the diameter of three resting lymphocytes), nuclear molding, growth in sheet-like patterns without delicate vascularization [30–47], but showed features of NETs such as oval nuclei (18/18, 100%), abundant cytoplasm, nested growth pattern and delicate vascularity (16/18, 89%). Of the remaining 2/20 patients with tumor images [48, 49], one had nodules in the upper and middle lobes of the right lung associated with prominent mediastinal lymph nodes, and the other had a large cavitary mass in the right lung and pleural effusion. The biopsy from a lymph node of the first patient was composed of highly pleomorphic, partly small and partly large NEC cells with a high Ki67 index of 80% and single cell positivity for ACTH [48]. The second patient’s biopsy was obtained from the lung mass [49] and the histologic image showed approximately 50 cells with pleomorphic nuclei and narrow cytoplasm and was therefore considered "compatible with SCLC." Of note in this group of 18 tumors that were reclassified as NETs are five tumors that had the histology of a spindle cell NET, which is known to be easily misdiagnosed as SCLC, especially on biopsy [50, 51].

In summary, our analysis of tumor images shows that in the majority of cases, they are not clearly indicative of SCLC, but of NET, and are therefore not suitable to support the diagnosis of SCLC as the cause of ECS. In the remaining 185 cases without histologic images or images of sufficient quality, we are also unable to make a definitive judgment on the accuracy of the diagnosis. This is a clear limitation of the study. However, the observation points out that the lack of histological documentation of the tumor is a deficiency that severely limits, if not nullifies, the significance of the respective case report as evidence of SCLC as the cause of ECS.

We were also unable to establish a diagnosis of SCLC in patients with ECS based on clinical description alone. Reported clinical features, such as disease stage, outcome, disease duration, and cause of death were of little or no help in distinguishing NEC from NET because most patients presented with extensive disease (93%) and complications (such as infections) of severe Cushing syndrome, that could not be reliably assigned to one or the other diagnosis. The same was true for the other clinical parameters such as sex, age, and smoking status. Taken together, the results of our systematic literature review suggest that the diagnosis of SCLC-based ECS is equivocal in the majority of cases and that the diagnosis of SCLC must be critically reconsidered and replaced by pulmonary NET (carcinoid).

In the second study, we pursued the question whether neoplastic ACTH-positive cells can arise in the lung and become a component of SCLCs not associated with ECS. Since we were unable to find any data on the occurrence of ACTH-positive cells in SCLCs without ECS in the literature, nor did we have any information on the presence of ACTH-positive cells in the normal lung [52], we screened a large series of resected SCLCs in whom no ECS was known. Our data in 155 resected SCLCs showed that only a small proportion of SCLCs (3%, 5/155) expressed ACTH. The ACTH-positive tumor cells were focal and showed weak positivity in individual cells (less than 5% of the tumor cells). The weak ACTH positivity may be explained by the rapid secretion of ACTH by these tumors. Another explanation could be a greatly reduced production of secretory hormone granules in these poorly differentiated tumor cells, leading to a loss of hormone storage capacity. The lower differentiation of SCLCs may also explain why in non-functioning pulmonary NETs ACTH-positive cells are much more frequent than in SCLCs (39% vs. 3%).

The few SCLCs with ACTH-positive cells did not differ from ACTH-negative SCLCs in size, cytology, Ki67 status, p53 expression or clinicopathological data. In the NET group, we noted the distinct differences. The NETs in the four patients with known ECS were characterized by the highest number of ACTH-positive cells per tumor. Moreover, these NETs differed in sex and age from those not associated with ECS, but did not differ in smoking status or other histopathological features. In particular, the ECS-associated NETs did not show NEC-like histological changes, which have recently been reported in mostly pancreatic NETs after prolonged multimodal therapy [53, 54]. Correlation of the number of ACTH-positive cells in ECS-negative tumors with serum ACTH levels in the corresponding patients was not possible because these patients were not monitored for serum ACTH preoperatively. This cross-sectional study precludes discussion of a dynamic expression of ACTH over time in SCLCs and NETs.

In conclusion, our literature review revealed that most (185/205) of the reported SCLC cases with ECS provided no or insufficient information for proper reassessment and confirmation of the morphological tumor diagnosis. Moreover, the 18 tumors for which histological images were available were most likely pulmonary NETs (carcinoids), leaving only 2 cases in which the diagnosis of SCLC could not be excluded and thus remained likely. Considering also the finding that single ACTH-positive cells were found in 3% of resected SCLCs, it seems that SCLC have the potential to develop ACTH producing tumors, but probably at a lower frequency than the reported cases suggest. In practice, this means that given the preponderance of pulmonary NETs as a cause of ECS, these tumors must be carefully excluded before accepting the diagnosis of SCLC.

## Supplementary Information

Below is the link to the electronic supplementary material.

Supplementary file1 (DOCX 24 KB)

Supplementary file2 (DOCX 17 KB)

Supplementary file3 (DOCX 16 KB)

Supplementary file4 (DOCX 18 KB)

Supplementary file5 (DOCX 18 KB)

Supplementary file6 (DOCX 21 KB)

Supplementary file7 (DOCX 108 KB)

## Data Availability

The Datasets used and analyzed during the current study are available from the corresponding author on reasonable request.
