# Supplementary material for: Is Ectopic Cushing Syndrome Commonly Associated with Small Cell Lung Cancer (SCLC)? Critical Review of the Literature and ACTH Expression in Resected SCLC
Source: Endocr Pathol. 2025 May 2;36(1):16. doi: 10.1007/s12022-025-09860-5 (PMC12048459; doi:10.1007/s12022-025-09860-5)
Supplement: Supplementary file 1 — Supplementary file1 (DOCX 24 KB) [file 12022_2025_9860_MOESM1_ESM.docx]

Supplementary Table 1: Number of patients with Ectopic Cushing syndrome associated with neuroendocrine neoplasms of pulmonary, pancreatic and gastrointestinal origins.

| ID | References | Total ^a^ (n) | Lung | | | Pancreas | | | GI | | |
| --- | --- | --- | --- | --- | --- | --- | --- | --- | --- | --- | --- |
| 1 | Davi et al. 2017 | 69 | Total | 49 (71%) |  | Total | 17 (25%) |  | Total | 3 (4%) |  |
|  |  |  |  | NET | 45 (92%) |  | NET | 17 (100%) |  | NET | 3 (100%) ^d^ |
|  |  |  |  | NEC | 4 (8%) ^b^ |  | NEC | 0 |  | NEC | 0 |
| 2 | Goroshi et al. 2016 | 8 | Total | 7 (88%) |  | Total | 1 (13%) |  | Total | 0 |  |
|  |  |  |  | NET | 6 (86%) |  | NET | 1 (100%) |  | NET | 0 |
|  |  |  |  | NEC | 1 (14%) ^b^ |  | NEC | 0 |  | NEC | 0 |
| 3 | Ejaz et al. 2011 | 24 ^c^ | Total | 18 (75%) |  | Total | 4 (17%) |  | Total | 1 (4%) |  |
|  |  |  |  | NET | 9 (50%) |  | NET | 4 (100%) |  | NET | 1 (100%) ^e^ |
|  |  |  |  | NEC | 9 (50%) |  | NEC | 0 |  | NEC | 0 |
| 4 | Isidori et al. 2006 | 24 | Total | 19 (79%) |  | Total | 3 (13%) |  | Total | 2 (8%) |  |
|  |  |  |  | NET | 12 (63%) |  | NET | 3 (100%) |  | NET | 0 |
|  |  |  |  | NEC | 7 (37%) ^b^ |  | NEC | 0 |  | NEC | 2 (100%) ^f^ |
| 5 | Ilias et al. 2005 | 46 | Total | 38 (83%) |  | Total | 1 (2%) |  | Total | 7 (15%) |  |
|  |  |  |  | NET | 35 (92%) |  | NET | 1 (100%) |  | NET | 7 (100%) ^g^ |
|  |  |  |  | NEC | 3 (8%) ^b^ |  | NEC | 0 |  | NEC | 0 |
| 6 | Aniszewski et al. 2001 | 15 | Total | 13 (87%) |  | Total | 2 (13%) |  | Total | 0 |  |
|  |  |  |  | NET | 1 (8%) |  | NET | 2 (100%) |  | NET | 0 |
|  |  |  |  | NEC | 12 (92%) ^b^ |  | NEC | 0 |  | NEC | 0 |
| 7 | Howlett et al. 1986 | 12 | Total | 9 (75%) |  | Total | 2 (17%) |  | Total | 1 (8%) |  |
|  |  |  |  | NET | 6 (67%) |  | NET | 2 (100%) |  | NET | 1 (100%) ^h^ |
|  |  |  |  | NEC | 3 (33%) ^b^ |  | NEC | 0 |  | NEC | 0 |

Footnote: Abbreviations: NEN, neuroendocrine neoplasm; NET, neuroendocrine tumor; NEC, neuroendocrine carcinoma; GEP, gastroenteropancreatic; Pan, pancreatic; GI, gastrointestinal; SCLC, small cell lung carcinoma, a) pulmonary and GEP NENs were extracted, b) exclusively SCLC, c) 1 GEP organ not further specified, d) 1 ileal, 1 cecal, and 1 rectal NET, e) 1 small intestinal, f) 2 colorectal small cell carcinomas, g) 1 appendix NET and 6 gastrinomas, h) gallbladder NET.
