# Supplementary material for: Is Ectopic Cushing Syndrome Commonly Associated with Small Cell Lung Cancer (SCLC)? Critical Review of the Literature and ACTH Expression in Resected SCLC
Source: Endocr Pathol. 2025 May 2;36(1):16. doi: 10.1007/s12022-025-09860-5 (PMC12048459; doi:10.1007/s12022-025-09860-5)
Supplement: Supplementary file 2 — Supplementary file2 (DOCX 17 KB) [file 12022_2025_9860_MOESM2_ESM.docx]

Supplementary Table 2: Keywords used for literature search.

| Organ | Tumor type | Functional parameters |
| --- | --- | --- |
| Bronchus | Oat cell | Cushing('s) syndrome |
| Bronchial | Small cell | ACTH |
| Bronchopulmonary | SCLC | Adrenocorticotropin |
| Chest | Large cell | Corticotropin |
| Lung | LCNEC | Ectopic |
| Mediastinal | NEC | Secrete hormone |
| Mediastinum | Neuroendocrine | Autoantibodies |
| Pulmonal | Carcinoid |  |
| Pulmonary | Tumorlets |  |

Footnote: Abbreviations: SCLC, small cell lung cell carcinoma; LCNEC, large cell neuroendocrine carcinoma; NEC, neuroendocrine carcinoma; DIPNECH, diffuse idiopathic pulmonary neuroendocrine cell hyperplasia; ACTH, adrenocorticotropic hormone. Keywords from each entity were combined using "AND" for PubMed search. "non-small" is used as an excluded keyword to avoid inclusion of non-small cell lung cancer. For example ((Bronchus) AND (oat cell) AND (ACTH) NOT (non-small))
