# Supplementary material for: Is Ectopic Cushing Syndrome Commonly Associated with Small Cell Lung Cancer (SCLC)? Critical Review of the Literature and ACTH Expression in Resected SCLC
Source: Endocr Pathol. 2025 May 2;36(1):16. doi: 10.1007/s12022-025-09860-5 (PMC12048459; doi:10.1007/s12022-025-09860-5)
Supplement: Supplementary file 3 — Supplementary file3 (DOCX 16 KB) [file 12022_2025_9860_MOESM3_ESM.docx]

Supplementary Table 3: Number of patients with pulmonary neuroendocrine neoplasms operated in each institution

|  | Institution | Country | SCLC | NET |
| --- | --- | --- | --- | --- |
| 1 | University Hospital rechts der Isar, Technical University Munich | Germany | 3 | 74 |
| 2 | University Hospital Regensburg | Germany | 6 | 16 |
| 3 | University Hospital Augsburg | Germany | 5 | 7 |
| 4 | Städtisches Klinikum München | Germany | 0 | 5 |
| 5 | Zentralklinik Bad Berka | Germany | 16 | 0 |
| 6 | Aomori Prefectural Central Hospital | Japan | 18 | 8 |
| 7 | Ishinomaki Red Cross Hospital | Japan | 12 | 4 |
| 8 | Iwate Prefectural Central Hospital | Japan | 19 | 11 |
| 9 | Iwate Prefectural Isawa Hospital | Japan | 11 | 3 |
| 10 | Miyagi Cancer Center | Japan | 14 | 8 |
| 11 | Miyagi Cardiovascular and Respiratory Center | Japan | 21 | 3 |
| 12 | Osaki Citizen Hospital | Japan | 3 | 0 |
| 13 | Sendai Medical Center | Japan | 3 | 4 |
| 14 | Tohoku Medical Pharmaceutical University | Japan | 15 | 2 |
| 15 | Tohoku University Hospital | Japan | 9 | 13 |
|  | Total |  | 155 | 158 |

Abbreviations: SCLC, Small cell lung cell carcinoma; NET, Neuroendocrine tumor
