# Supplementary material for: Is Ectopic Cushing Syndrome Commonly Associated with Small Cell Lung Cancer (SCLC)? Critical Review of the Literature and ACTH Expression in Resected SCLC
Source: Endocr Pathol. 2025 May 2;36(1):16. doi: 10.1007/s12022-025-09860-5 (PMC12048459; doi:10.1007/s12022-025-09860-5)
Supplement: Supplementary file 4 — Supplementary file4 (DOCX 18 KB) [file 12022_2025_9860_MOESM4_ESM.docx]

Supplementary Table 4. Detailed information of immunohistochemical stainings

| Antibody | Source | Clone/ Catalog number | Dilution | Product description |
| --- | --- | --- | --- | --- |
| Synaptophysin | Invitrogen, Carlsberg, USA | SP11 | 1:50 | Rabbit, monoclonal |
| Chromogranin A | Abcam, Cambridge, UK | ab15160 | 1:300 | Rabbit, polyclonal |
| CD56 | Merck, Darmstadt, Germany | MRQ-42 | 1:200 | Rabbit, monoclonal |
| CK18 | Sigma-Aldrich Chemie GmbH, Taufkirchen, Germany | CY-90 | 1:2000 | Mouse, monoclonal |
| Ki-67 | Dako, Glostrup, Denmark | MIB-1 | 1:50 | Mouse, monoclonal |
| TTF-1 | Zytomed Systems, Berlin, Germany | 8G7G3/1 | 1:50 | Mouse, monoclonal |
| SSTR2 | Abcam, Cambridge, UK | ab134152 | 1:150 | Rabbit, monoclonal |
| p53 | Dako, Glostrup, Denmark | DO-7 | 1:200 | Mouse, monoclonal |
| Rb1 | BD Biosciences, Michigan, USA | G3-245 | 1:100 | Mouse, monoclonal |
| ACTH | Dako, Glostrup, Denmark | 02A3 | 1:2000 | Mouse, monoclonal |

Abbreviations: ACTH, adrenocorticotropic hormone; Rb1, retinoblastoma 1; SST2, somatostatin receptor 2; TTF-1, thyroid transcriptional factor-1
