# Supplementary material for: Is Ectopic Cushing Syndrome Commonly Associated with Small Cell Lung Cancer (SCLC)? Critical Review of the Literature and ACTH Expression in Resected SCLC
Source: Endocr Pathol. 2025 May 2;36(1):16. doi: 10.1007/s12022-025-09860-5 (PMC12048459; doi:10.1007/s12022-025-09860-5)
Supplement: Supplementary file 5 — Supplementary file5 (DOCX 18 KB) [file 12022_2025_9860_MOESM5_ESM.docx]

Supplementary Table 5: Available clinical features of reported small cell lung carcinoma patients with ectopic Cushing syndrome

|  | Patients with available data | Patients with missing data | Data provided | |
| --- | --- | --- | --- | --- |
| Total N (%) | 205 (100) |  |  |  |
| Age | 187 (91) | 18 (9) | Median (range) | 58 (22-81) |
| Sex | 188 (92) | 17 (8) | Male: Female | 124:64 |
| Size (mm) | 32 (16) | 173 (84) | Median (range) | 43 (4-120) |
| Mitotic counts | 1 (0.5) | 204 (99.5) | Median (range) | 80 (80) |
| Necrosis | 4 (2) | 201 (98) | Diffuse (%) | 1 (25) |
|  |  |  | Focal (%) | 3 (75) |
| Ki-67 LI (%) | 3 (1) | 202 (99) | Median (range) | 80 (80-100) |
| Disease stage | 137 (67) | 68 (33) | Limited stage N (%) | 10 (7) |
|  |  |  | Extended stage N (%) | 127 (93) |
| Smoking | 66 (33) | 138 (67) | Smoker N (%) | 66 (100) |
|  |  |  | Non-smoker N (%) | 0 |
