# Supplementary material for: Is Ectopic Cushing Syndrome Commonly Associated with Small Cell Lung Cancer (SCLC)? Critical Review of the Literature and ACTH Expression in Resected SCLC
Source: Endocr Pathol. 2025 May 2;36(1):16. doi: 10.1007/s12022-025-09860-5 (PMC12048459; doi:10.1007/s12022-025-09860-5)
Supplement: Supplementary file 6 — Supplementary file6 (DOCX 21 KB) [file 12022_2025_9860_MOESM6_ESM.docx]

|  |  | | | | | SCLC | NET |
| --- | --- | --- | --- | --- | --- | --- | --- |
| Total | | N | | |  | 155 | 158 |
| Age ^a^ | |  | | Median (range) | | 69 (43-86) | 67 (11-86) |
| Sex ^a^ | | N (%) | M : F | | | 120 (77): 23 (23) | 55 (35): 103 (65) |
| Smoking ^b^ | | N (%) | Smoker | | | 102 (97) | 32 (44) |
|  |  |  | Never smoker | | | 3 (3) | 40 (56) |
| Ki-67 index | | % | Median (range) | | | 75 (25-99) | 2 (0.2-62) |
| NET classification | | N (%) | TC : AC | | | - | 124 (78) : 34 (22) |
| p53 status ^c^ | | N (%) | Normal | | | 49 (37) | 141 (100) |
|  |  |  | Abnormal | | | 84 (63) | 0 (0) |
| RB1 status ^d^ | | N (%) | Normal | | | 13 (89) | 124 (95) |
|  |  |  | Abnormal | | | 101 (11) | 7 (5) |
| SSTR2 ^e^ | | N (%) | Negative | | | 58 (56) | 63 (48) |
|  |  |  | Positive | | | 45 (44) | 67 (52) |
| ACTH | | N (%) | Score 0 | | | 150 (97) | 97 (61) |
|  |  |  | Score 1 | | | 5 (3) | 30 (19) |
|  |  |  | Score 2 | | | 0 (0) | 21 (13) |
|  |  |  | Score 3 | | | 0 (0) | 10 (6) |
| Cushing syndrome | | N (%) | Absent | | | 155 (100) | 154 (97) |
|  |  |  | Present | | | 0 (0) | 4 (3) |

Supplementary table 6: Clinicopathological characteristics of lung neuroendocrine neoplasms examined for immunohistochemical ACTH expression

Footnote: Abbreviations: SCLC, Small cell lung cell carcinoma; NET, Neuroendocrine carcinoma; M, male; F female; TC, Typical carcinoid; AC, Atypical carcinoid, RB1, Retinoblastoma 1; SSTR2, Somatostatin Receptor 2. Data missing in a)12 SCLC patients, b) 50 NET and 86 SCLC patients, c) 22 SCLC and 17 NET patients, d) 41 SCLC and 27 NET patients, e) 52 SCLC and 29 NET patients.
