# Supplementary material for: Is Ectopic Cushing Syndrome Commonly Associated with Small Cell Lung Cancer (SCLC)? Critical Review of the Literature and ACTH Expression in Resected SCLC
Source: Endocr Pathol. 2025 May 2;36(1):16. doi: 10.1007/s12022-025-09860-5 (PMC12048459; doi:10.1007/s12022-025-09860-5)
Supplement: Supplementary file 7 — Supplementary file7 (DOCX 108 KB) [file 12022_2025_9860_MOESM7_ESM.docx]

Supplementary Figure 1: Literature search algorithm for small cell lung carcinoma (SCLC) with ectopic Cushing syndrome (ECS).


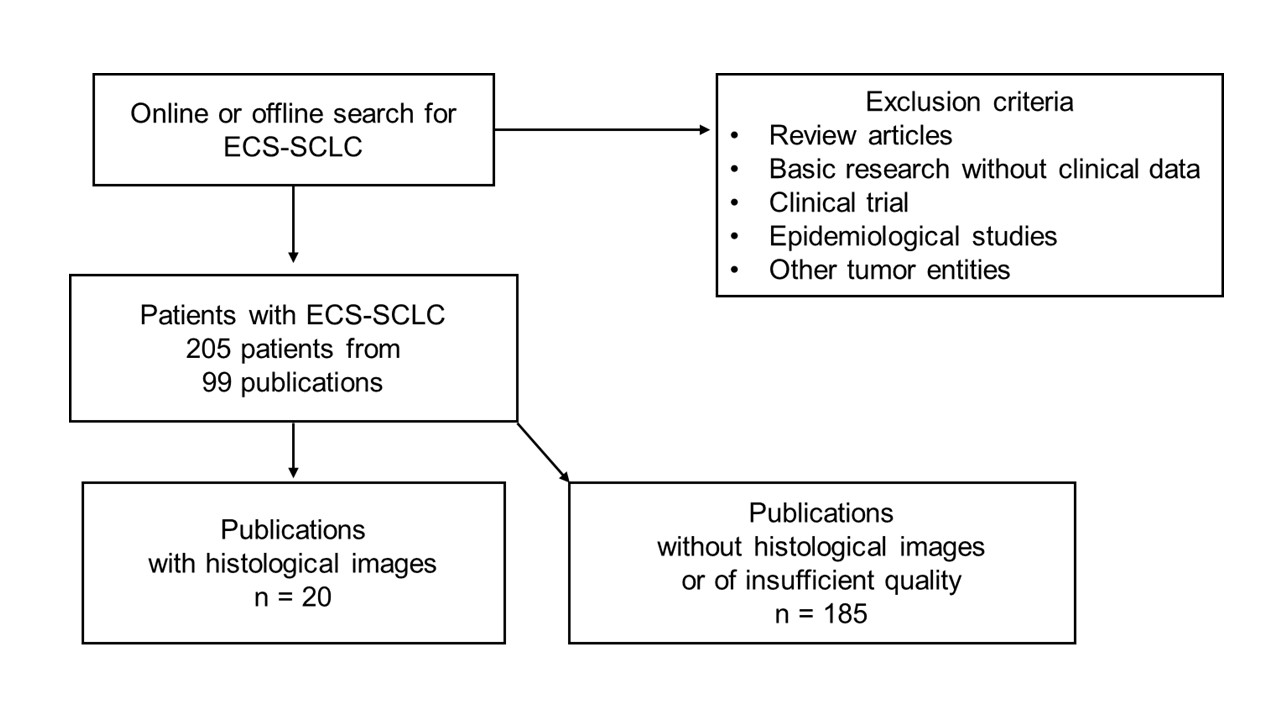


Endocrine Pathology, A. Ura et al. Department of Pathology. Technical University of Munich, TUM school of Medicine and Health, Munich, Germany, atsuko.kasajima@tum.de
